# RpoB H481Y Rifampicin Resistance Mutation‐Associated Oxidative Stress Sensitivity Reduces the Virulence of *Staphylococcus aureus*

**DOI:** 10.1111/1348-0421.13190

**Published:** 2024-12-01

**Authors:** Tomonori Kano, Kazuya Ishikawa, Lina Imai, Kazuyuki Furuta, Chikara Kaito

**Affiliations:** ^1^ Graduate School of Medicine, Dentistry and Pharmaceutical Sciences Okayama University Okayama Japan

**Keywords:** oxidative stress, RNA polymerase beta subunit, *Staphylococcus aureus*, virulence

## Abstract

In this study, we have established a *Staphylococcus aureus* RpoB H481Y mutant strain and demonstrated that it is sensitive to menadione or hydrogen peroxide‐induced oxidative stress and exhibits reduced virulence against a silkworm infection model. Furthermore, the reduced virulence of the RpoB H481Y mutant was abrogated in the presence of *N*‐acetylcysteine, a reactive oxygen species scavenger. These results suggest that oxidative stress sensitivity caused by the RpoB H481Y rifampicin resistance mutation attenuates the virulence of *S. aureus*.

**Abbreviations**
PBS: phosphate buffered saline
ROS: reactive oxygen species
TSB: tryptic soy broth

*Staphylococcus aureus* is a human pathogenic bacterium that causes various diseases including pneumonia, meningitis, and sepsis. Its high adaptive capacity to the host environment and the development of antibiotic resistance complicate staphylococcal therapeutics [1]. Therefore, understanding the molecular mechanisms that regulate these factors is crucial for the effective treatment of staphylococcal infections.

Rifampicin binds to RpoB, an RNA polymerase beta subunit [2]. Amino acid substitutions in RpoB induce rifampicin resistance in bacteria. Some RpoB mutations lead to reduced sensitivity to β‐lactam antibiotics and vancomycin in *S. aureus* [3, 4, 5]. The RpoB H481Y mutation is one of the most prevalent rifampicin resistance‐associated mutations in *S. aureus* [6]. Previously, the RpoB H481Y mutation in *S. aureus* was reported to cause reduced virulence in a murine bacteremia model [7] and a *Galleria mellonella* infection model [8]; however, the underlying mechanism remains unclear. In this study, we focused on the previous finding that the *S. aureus* RpoB H481Y mutation induces sensitivity to menadione, a drug that produces oxidative radicals [9] and hypothesized that the RpoB H481Y mutation‐associated oxidative stress sensitivity potentially causes the reduced virulence of *S. aureus*.

We obtained a rifampicin‐resistant mutant carrying the RpoB H481Y mutation by culturing the *S. aureus* RN4220 strain in the presence of rifampicin. To eliminate unintended mutations other than the RpoB H481Y mutation, the RpoB H481Y mutation was transferred to a wild‐type (WT) strain by transduction using the phage 80α. The suicide plasmid pCK20 [10] carrying a chloramphenicol resistance gene was integrated into the upstream region of the RpoB H481Y mutation, which is a *sigH*‐*secE* intergenic region, *via* homologous recombination and the chloramphenicol resistance was used as a selection marker. The transductant carrying the RpoB H481Y mutation was used as a RpoB H481Y mutant in subsequent experiments. Moreover, we constructed a control strain, the RpoB WT strain, by integrating the pCK20 into the upstream region of *rpoB* in the WT strain. The primers, bacterial strains, and plasmids used in this study are listed in Tables 1 and 2. Rifampicin resistance in the RpoB H481Y mutant was confirmed by culturing it on tryptic soy agar plates (TSB agar plates) containing rifampicin (Figure 1A).

**Table 1:** Primers used in this study.

Primers used in the study	
secE‐up‐F	GGTCGGTCCAAACATTTGAT
secE‐up‐R	TTGTGTGCGCGTTACATTTT

**Table 2:** Bacterial strains and plasmids used in this study.

Strain or plasmid		Source or reference
Strains		
RN4220	NCTC8325‐4, restriction mutant	[11]
TK0002	RN4220 *sigH*‐*secE* intergenic region::pCK20; Cm^r^, *rpoB* WT	This study
TK0003	RN4220 *sigH*‐*secE* intergenic region::pCK20; Cm^r^, *rpoB* H481Y	This study
JM109	*Escherichia coli* host strain for cloning	Takara Bio
Plasmids		
pCK20	*S. aureus* suicide vector for targeting; Cm^r^	[10]

**Figure 1:**
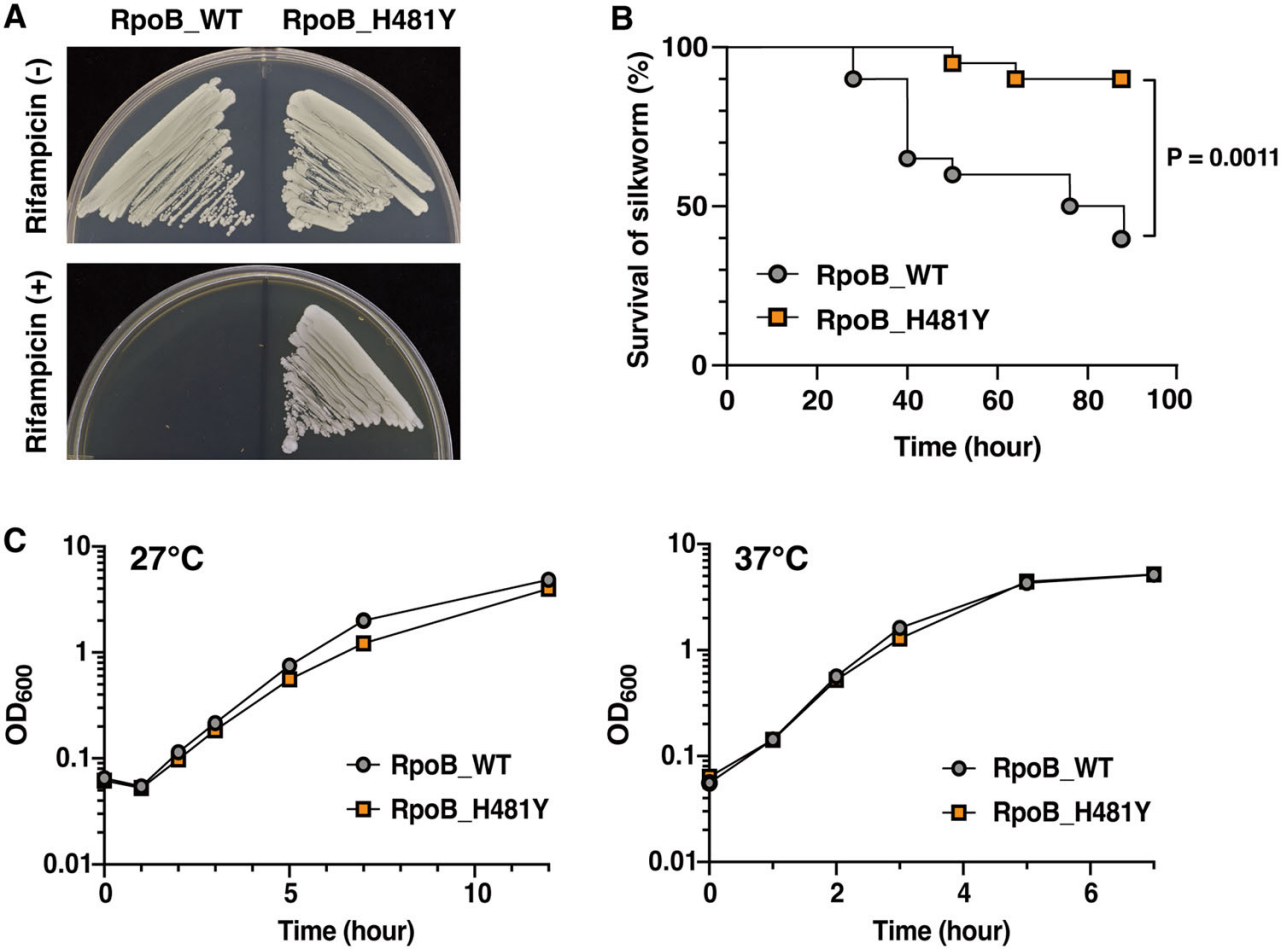
RpoB H481Y mutation attenuates the virulence of *Staphylococcus aureus* in the silkworm infection model. (A) The rifampicin resistance of the RpoB_H481Y mutant was examined. Overnight grown bacterial cultures of RpoB_WT (left) and RpoB_H481Y (right) were streaked on TSB agar plates supplemented with 50 µg/mL rifampicin and incubated overnight at 37°C. (B) The silkworm‐killing activity of the RpoB_H481Y mutant *S. aureus* was examined. Silkworms (*n* = 20) were injected with *S. aureus* cells (1.3 × 10^7^ CFU) and the survivals were monitored at 27°C. Log‐rank test *p*‐values are presented. (C) Growth curves of RpoB_WT and RpoB_H481Y strains at 27°C and 37°C were examined. Overnight grown cultures were inoculated into fresh TSB medium and cultured at 27°C or 37°C with shaking. OD_600_ was measured using a spectrophotometer at the indicated time points. Data are presented as the means ± SD of three biological replicates.

The virulence of the RpoB H481Y mutant was evaluated using a silkworm infection model [12, 13]. Silkworms injected with the RpoB H481Y mutant exhibited a higher survival rate than the RpoB WT‐injected group throughout the observation period (Figure 1B). However, the growth curve did not considerably differ between the RpoB H481Y mutant and RpoB WT strains at 27°C (Figure 1C), the temperature maintained during the silkworm infection analysis, and at 37°C (Figure 1C). These results suggest that the RpoB H481Y mutation attenuates *S. aureus* virulence in silkworms.

To validate the previously reported RpoB H481Y mutation‐induced sensitivity to menadione in *S. aureus* [9], we studied the RpoB H481Y mutant colony formation on a TSB agar plate containing menadione; the RpoB H481Y mutant formed fewer colonies than the RpoB WT strain, whereas, the number of colonies of both strains were approximately comparable in the absence of menadione (Figure 2A). To examine the sensitivity of the RpoB H481Y mutant to oxidative stress, we performed a bactericidal assay in the presence of hydrogen peroxide; the RpoB H481Y mutant exhibited a lower survival rate than the RpoB WT strain after treatment with hydrogen peroxide (Figure 2B), suggesting a RpoB H481Y mutation‐induced oxidative stress sensitivity in *S. aureus*.

**Figure 2:**
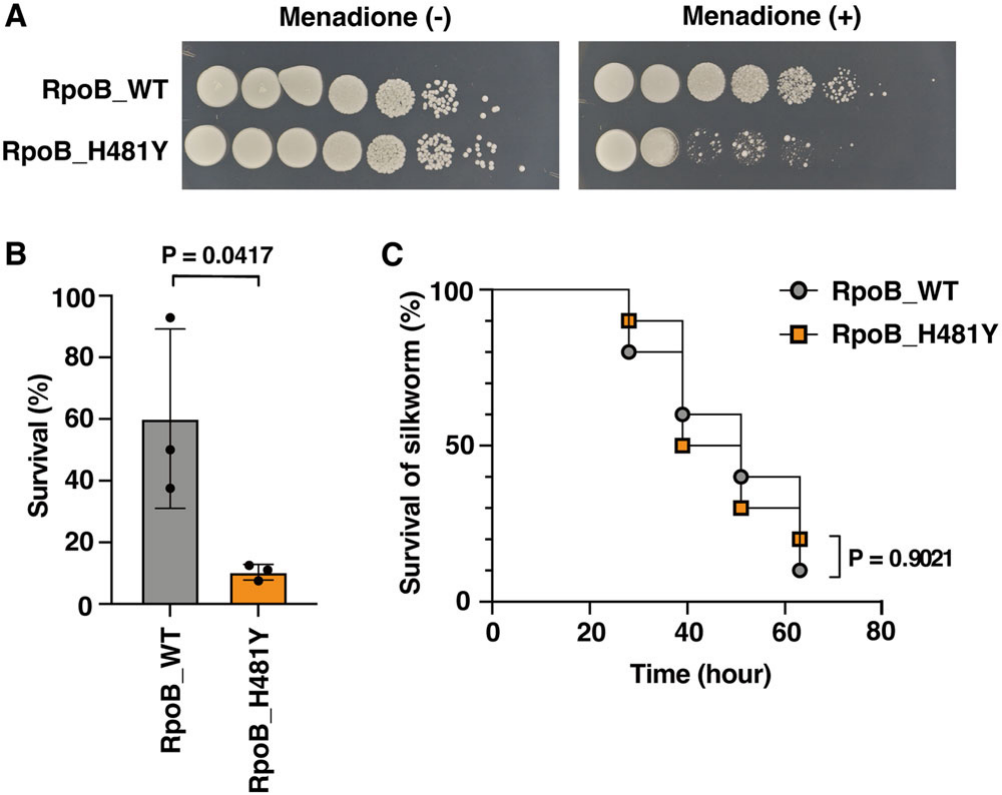
The attenuated virulence of *Staphylococcus aureus* RpoB H481Y mutant is abolished in the presence of *N*‐acetylcysteine. (A) Overnight grown cultures of RpoB_WT and RpoB_H481Y strains were serially diluted 10‐fold using phosphate‐buffered saline (PBS); 8 µL were spotted onto TSB agar plate supplemented with 36 µM menadione and cultured overnight at 37°C. (B) Overnight grown cultures of RpoB_WT and RpoB_H481Y strains were used to inoculate fresh TSB medium and incubated at 37°C to achieve OD_600_ = 0.5. Cells were adjusted to 1 × 10^8^ CFU/mL using PBS and treated with 4.4 mM H_2_O_2_ for 15 min at 37°C. Subsequently, cells were washed by PBS and serially diluted 10‐fold; 8 µL were spotted onto TSB agar plates, cultured overnight at 37°C, and Colony Forming Units were investigated. Survival rates were calculated as a ratio of the data acquired from the untreated group. Data are presented as the means ± SD of three biological replicates. Statistical analysis was conducted using Student's *t* test. (C) The silkworm‐killing activities of the RpoB_WT and RpoB_H481Y strains in the presence of N‐acetylcysteine were evaluated; silkworms (*n* = 10), pretreated with 2.5 mg *N*‐acetylcysteine, were injected with bacterial cells (1.3 × 10^7^ CFU), and the survivals were monitored at 27°C. Log‐rank test *p*‐values are presented.

The innate immune response involves phagocytes that utilize oxidative stress to eliminate pathogens [14]. Oxidative stress plays a vital role in the immunity of silkworms against *S. aureus* [15, 16, 17]. To assess whether the attenuated virulence of the RpoB H481Y mutant was associated with oxidative stress sensitivity, we infected silkworms with the RpoB H481Y mutant in the presence of the reactive oxygen species (ROS) scavenger *N*‐acetylcysteine and revealed no significant difference in silkworm‐killing activity between the RpoB H481Y mutant and the RpoB WT strain (Figure 2C). This result suggests that oxidative stress sensitivity caused by the RpoB H481Y mutation is responsible for attenuated virulence.

In this study, we demonstrated that the RpoB H481Y rifampicin resistance mutation in *S. aureus* reduced virulence against a silkworm infection model, which is consistent with previously reported data derived from other animal infection models, such as mice [7] or *G. mellonella* larvae [8]. Furthermore, the RpoB H481Y mutant exhibited oxidative stress sensitivity, and its reduced killing activity was abrogated by a ROS scavenger. This is the first report of RpoB H481Y rifampicin resistance mutation‐induced oxidative stress sensitivity, which causes reduced virulence in *S. aureus*.

However, the mechanism underlying RpoB H481Y mutation‐mediated induction of oxidative stress sensitivity remains unclear. *S. aureus* RpoB H929P or Q645H mutations cause transcriptional dysfunction, promoting intracellular accumulation of ribonucleotides [4]. In contrast, some RpoB mutant strains, including *Escherichia coli* H526P, exhibit increased transcription termination read‐through [9, 18], which can decrease the ribonucleotide pool. As *S. aureus* RpoB H481 corresponds to *E. coli* RpoB H526 and ROS targets and destroys ribonucleotides, the RpoB H481Y mutation possibly increases transcription termination read‐through, reduces the ribonucleotide pool, and weakens the reduced nucleotide pool in response to oxidative stress. Nucleotide pools are crucial for repairing DNA double‐strand breaks caused by oxidative stress or quinolone antibiotics [19, 20]. Further investigation will illustrate the molecular mechanism underlying oxidative stress sensitivity caused by the RpoB H481Y mutation.

## Disclosure

Chikara Kaito is the Editor‐in‐Chief of Microbiology and Immunology and a co‐author of this article. They were excluded from editorial decision‐making related to the acceptance and publication of this article.

## Ethics Statement

The authors have nothing to report.

## Data Availability

The authors have nothing to report.
